# A Systematic Treatise, Historical, Etiological, and Practical, on the Principal Diseases of the Interior Valley of North America, as They Appear in the Caucasian, African, Indian, and Esquimaux Varieties of Its Population

**Published:** 1850-09

**Authors:** 


### REVIEWS.

---

Art. II.—*A Systematic Treatise, Historical, Etiological, and Practical, on the Principal Diseases of the Interior Valley of North America, as they appear in the Caucasian, African, Indian, and Esquimaux varieties of its Population.* By Daniel Drake, M. D. Cincinnati: Winthrop B. Smith & Co. Philadelphia: Grigg, Elliott & Co. New York: Mason & Law. 1850.

We have already announced the publication of this work and briefly expressed the high estimate we have formed of its merits. The most cursory examination is sufficient to show that it is the fruit of more patient investigation, of more mental and bodily toil, of more extensive and varied research than are often devoted to books at the present day. The work indeed has been long on hand. Forty years ago, while a young man and still an undergraduate in medicine, the author published the germ of it in a pamphlet entitled "Notices Concerning Cincinnati;" an effort which gave proof of those fine powers of mind which are so remarkably displayed in the volume before us. As long ago as thirty years, Dr. Drake informs us in his preface, he "formed the design of preparing a more extended work on the diseases of the Ohio valley;" and not long afterwards his purpose was made known to the public. During the years that have since elapsed, although much engaged in the practice of medicine and engrossed by the politics of his profession, he has never lost sight of his early enterprise. Amid all the revolutions of medical schools in which he has borne a conspicuous part, his plans in regard to this great labor have remained unchanged. The "image" of the institution founded in his youth, in his favorite city, and by his efforts, which has pursued him in all his journeyings, and haunted and made him restless in all other institutions—an image beckoning him still to return to his early home and still disappointing him—has never succeeded in diverting his thoughts wholly from his cherished work. Fortunately for his fame and the interests of medical science, amid every distracting care, and through the unquiet life of a medical politician and pioneer, this noble object has been kept steadily before his mind.

It was about nine years ago that Dr. Drake set seriously by travel and personal observation and inquiry about collecting the materials for his treatise. Connected at the time with the medical school at Louisville, he enjoyed in its long vacations the leisure requisite to the tedious journeys which the plan of his work rendered necessary. In the spring of 1841 he commenced a series of excursions which were continued through a number of years. Early in that year he descended the Mississippi to New Orleans, crossed over to the bays of Mobile and Pensacola, and inspected the medical topography of those interesting regions. In the ensuing spring he made a second voyage down the Mississippi, on this occasion extending his observations as far as the Balize. Much time was spent in the city of New Orleans collecting facts relative to the diseases to which that great Southern emporium is subject, and, returning, he penetrated into the State of Mississippi in various directions, as, during the former year, he had done into Alabama. Later in the season he ascended the Tennessee, Ohio, Missouri, and upper Mississippi rivers far towards their sources, and inspected the localities on each most interesting to the medical topographer. From year to year similar excursions were made until he had taken a general survey of the great valley, from the mouth of the Mississippi to the mouth of the St. Lawrence, and from Pensacola Bay to Lake Superior. It was an object with the author to converse with medical men, as well as to see medical topography, and to collect from them the results of their observations in reference to the diseases of their several countries, thus rendering his work a summary of the professional experience of the interior valley.

From this narrative it will be seen, that Dr. Drake has laid the best possible foundation for such a work as the one he has long meditated. He has seen the country, the diseases of which he is to describe, in all its aspects and in every stage of culture and civilization. He was careful to visit all the barracks in his line of travel, where he might see men congregated in numbers, and collect the most reliable statistics relative to their diseases; he visited the plantations in Alabama, Mississippi, and Louisiana for the purpose of learning what there was peculiar in the complaints of the unmixed African race; and he also went among some of the Indian tribes to observe how far disease is modified by the primitive habits of savage life. In addition to this, he has drawn from the observations of other travelers in remote parts of the valley not explored by himself, so that his treatise will be found to embrace a description of the medical topography of the country, from Vera Cruz to the regions around Hudson Bay.

The field comprehended in this survey is an immense and very remarkable one. The interior valley of North America spreads through fifty degrees of longitude and forty of latitude, from the Appalachians to the Rocky mountains, and from the Gulf of Mexico to the Polar Sea. It embraces every variety and extreme of climate, of surface, of geological formation, and, among its inhabitants, nearly all the varieties of the human race. Over a wide space its surface is but little raised above the level of the ocean, but it is overlooked by mountains which pierce the line of perpetual congelation. Broad regions are covered by ponds and marshes, while interminable planes of arid sand are spread out over other portions of it. Beginning at the Gulf with the most recent alluvion, its formations run through every geological era up to the primitive rocks. Stretching from zone to zone, it has the temperature of the tropics and of the arctic circle. Many thousand miles of its surface reposing under a tropical sun are hardly above the tides of the Gulf, but advancing North it presents an Alpine region extending through many degrees of latitude, with a mean elevation of more than two thousand feet above the level of the sea. It is traversed by the longest river in the world, and contains the most extended chain of lakes. Within its bosom are found the Falls of Niagara and the Mammoth Cave.

A picture of so vast and diversified a region could hardly fail to prove interesting, and, if faithfully and ably executed, must be interesting and instructive to medical men in an uncommon degree. At the same time, to take in all the physical features of such a country, to compare its topography in different latitudes and under all the circumstances which influence disease, to determine the laws of its climate and the conditions under which its endemic disorders arise, and finally to give an accurate description of those disorders and the most successful mode of treating them, will be admitted by all to be a task of no ordinary magnitude. This is the task which Dr. Drake, during the greater part of his life, has had in contemplation, upon which he has entered in the volume before us, and has accomplished, as we have already said, with eminent success. It was to present a work on the diseases of the interior valley the materials of which should be collected in the valley itself, that he has been so long making preparation. It was never any part of his scheme to compile a treatise out of the labors of other authors. It has been his design to give in his book what he has himself observed and tried, and what has been sanctioned by the experience and fallen under the observation of the physicians resident in the country to which his work relates. With such a purpose, it was to be expected that his production would contain much that was peculiar and distinctive; not only much that was new in matter, but something original in its plan and arrangement. It was to have been expected that it would depart widely from the ordinary systematic treatises, which every year adds to the stock of our literature on the practice of medicine, and a little examination will prove that such is the fact. We propose to point out some of its most characteristic features.

The first of these that we shall notice is the attention given by Dr. Drake to the relations of geology to medicine. In the numerous pages of his work strictly introductory to the consideration of special diseases, he has constantly indicated these relations. Very justly he regards mineral geology as the proper basis of medical topography, and he is the first systematic writer on medicine who has given prominence to this fact. Upon the nature of the surface of a country is well understood to depend, in a material degree, the health of its inhabitants, or the reverse, and the character of its surface is dependent upon the stratum of rocks upon which it rests. Where the formation is a sandstone, the country is rolling, hilly, mountainous, comparatively arid, and sterile; its streams are clear and have good currents, and it is free from ponds and stagnant water. On the other hand, where clayslate constitutes the substratum, the land is level and retentive of moisture, in consequence of which accumulations of water occur. It is the region of ponds and marshes—the region of intermittent fevers. A limestone formation is the one most favorable to fertility of soil, and is found as the basis of countries remarkable for their fruitfulness. The surface, where it constitutes the prevailing rock, is covered with a luxuriant vegetation, and if clay enter freely into the composition of the superincumbent soil, ponds are formed. There are local exceptions to these rules, but such will be found to be the general law. Its bearing upon etiology need not be pointed out. When the physician is informed that the geological structure of a country is marked by one or the other of these characters he will be able at once to say what are its chances for health. All medical experience goes to the point, that countries in which sandstone is the prevailing rock are healthy—that districts resting upon slate, temperature and other circumstances favoring, are subject, especially, to fever and ague—and that rich limestone regions are the favorite abodes of autumnal fevers.

Geology is a science of modern origin. A few years ago it was in an embryonic condition. Its vocabulary was not settled and it was without system. But light and order have been infused into it by philosophers still living; its language has become precise and intelligible; great leading principles have been fully established and are now universally recognised and accredited. A parallelism has been traced between the formations of Europe and those of North America, and now when a geologist in Indiana or Kentucky alludes to a particular system, he is as well understood by his brethren in London or Paris as if he were writing to them from one of the neighboring counties or provinces. The strata which compose the earth have a regular order of sequence which is never reversed. The system which lies at the bottom in Russia is the foundation upon which the various systems repose in New York and Tennessee. A particular series may be absent, but it is never dislocated, unless by violence, as in the case of mountains where the strata are occasionally found displaced and contorted in all directions.

The geology of the great intermontane plane of North

America is marked by great simplicity; its formations are few and well defined. The primitive and metamorphic rocks—granite, gneiss, porphyry, mica-schist, syenite, &c.—are seen in a few places only, brought to the surface by upheaval, and upon these rest the lower Silurian series of rocks, to which succeed with great regularity the upper Silurian, the Devonian, and the Carboniferous, with which, over a wide district of our continent, the formations close. In some places the Devonian strata thin out until they can hardly be recognised; in some, one member, and in others another of the Carboniferous system is wanting. To the Devonian, in some regions, the Chalk formation succeeds, and over a wide tract the Drift formation rests upon Silurian strata, the carboniferous series being absent. But the particular series is easily made out by any one slightly acquainted with paleontology wherever the rocks come to the surface.

As a general, almost universal rule, the Silurian rocks of the interior valley are limestone, which, however, varies much in its lithological characters, in some cases being hard, compact, and durable, while in others it is soft, friable, and readily disintegrated. In one place the same series of limestones, containing the same fossils and belonging evidently to the same geological age, are readily worn down by the weather and have formed a deep, rich soil, and in another are so solid that they yield scarcely at all to the chemical and physical action of the atmosphere, and are scarcely covered with the most scanty soil. Perhaps no other rock is so variable in its minerological character as the limestone.

The Devonian system embraces limestones and clay-slate. This series thins out towards the South, but attains to great thickness in the North-western part of the valley.

The Carboniferous system embraces every variety of rocks—sandstones, limestones, and conglomerate—and, as the name imports, is the series in which bituminous coal is found. It begins with a fine grained sandstone, and ends with the coal series. The limestone of this system is that which forms the substratum of the barrens in Kentucky, and is the one in which the Mammoth Cave occurs.

Dr. Drake, in his medical topography, refers continually to this classification of strata and notes everywhere the formation constituting the surface-rock, specifying, at the same time, its mineralogical character; so that we know, when we have finished his description of a place, not only the geological age of the country, but whether its beds of stone are sandy, slaty, or calcareous. As the nature of the water is determined by the mineral strata over which it runs, we have only to inquire whether the prevailing rock at any point be sandstone, slate, or limestone, and we are at once informed whether the water is pure, or impregnated with mineral matters. Here we have the *telluric* element concerned in the elimination of diseases, and it has never anywhere else been as distinctly and fully recognised and as clearly pointed out as in this treatise.

To appreciate the bearing of mineral geology upon etiology, one has only to review the progress of cholera through the United States, either in its former or its present invasion, in both of which it will be found to have been remarkably under the influence of telluric as well as climatic agencies. At Quebec and Montreal, where the epidemic first touched our continent in 1832, the formation belongs to the lower Silurian. The same formation, together with limestones of a later era, prevails throughout a large portion of the State of New York, over which the pestilence swept with great violence. St. Louis is built upon carboniferous limestone. At Cincinnati, Lexington, Versailles, Nashville, Shelbyville and Lebanon, Tennessee, the lower Silurian limestone is the surface rock. At other places in the valley of the Mississippi where cholera has been most malignant, as at Sandusky and other towns in Ohio and in Indiana, the formation is of the Devonian era, but is still a limestone. In a word, limestone forms the great pan of the valley, and it is known that the epidemic has prevailed with a greater fatality here than anywhere else in our country. Except at New Orleans, the towns in Mississippi and Louisiana, and the cities on our Atlantic border, limestone, we believe, constitutes the basis of every soil in which cholera has revelled on the northern part of our continent. So far as our knowledge extends, it has no where prevailed with virulence in a sandstone district. Its chosen seats appear to have been limestone and alluvial soils.

We wish it to be distinctly understood that we do not mean, in saying this, to intimate a belief that limestone—much less that *limestone water*—is the cause of cholera. We do not allow the slightest weight to this hypothesis, but the facts to which we have referred have their value, and cannot be overlooked in our search after the cause of the epidemic. This telluric agency is one of the factors, and can be no more omitted in the estimate than the climatic. The earth and the atmosphere both seem concerned in the elimination of the peculiar poison; they furnish the two elements necessary to the result.

After all that we have said we need hardly add, that we consider this feature in Dr. Drake's Treatise a very important one—one possessing practical as well as philosophical interest—and that in thus developing the connection between etiology and geology, the author has set an example worthy of the imitation of future writers on medical topography. Indeed, we feel satisfied that we are only at the beginning of this subject, and that valuable truths will soon be brought to light in relation to it.

We remark further, in reference to the characteristics of this work, that it comprises the most extended and systematic view of medical topography with which our reading has made us acquainted. The author has not only described the various localities in the great interior valley, but shown their relations to each other, reducing all the parts of this wide hydrographical basin to a unit. The advantages of such a system, over the detached pieces of topography such as have been generally attempted, will appear at once. The reader, when he has completed Dr. Drake's description of the valley, has all its diversified features before him as a whole. He is able to compare its extreme southern, with its middle and extreme northern regions, in respect to disease, and to estimate the influence of elevation upon its endemic disorders. Starting on the shores of the Gulf and at the level of the sea, he may trace the fevers of the country through its changing climates, as it stretches towards the arctic circle and rises above the ocean, until they finally disappear under the rigorous skies of the north. He sees its southern, in contrast with its eastern and northern basins, the valley of the Mississippi, in contrast with the valley of the St. Lawrence, and its low, paludal planes, in contrast with its alpine regions. He has at once under his eye the point of maximum cold for the continent, and valleys swept by warm breezes from the Carribean sea. In short, he has a map of the medical topography of this remarkable region spread out before him—a map to which he will recur, again and again, with never failing interest.

The climate of the valley of the Mississippi has attracted the attention of several philosophers. The celebrated traveler, Volney, from a few brief observations, concluded that it was characterised by a mysterious mildness; and Mr. Jefferson also states that on the same parallel it is warmer than the Atlantic side of the Alleghany, the reverse of which is the fact. Dr. Drake enters with much minuteness into the discussion of the subject. Climate, as we have said, is one of the elements concerned in evolving the causes of disease, and is therefore a subject of the highest interest and importance to the physician. The soil of itself is not sufficient to develop the poison, however well adapted to it; climate of itself is not capable of generating it—the two must conspire. Arid planes of sand are healthy under the intense fervor of a tropical sun, and in latitudes far to the north marshes lose their pestilential attributes, numerous instances of which are cited in this treatise; the fact, however, required no additional confirmation, for it has long rested upon the common observation of men. But the general study of climate is not sufficient for the practitioner of medicine—he must study the climate to which he and his patients are exposed; for we need hardly say that every country has its own peculiar climate, the laws of which must be determined for themselves. The elements which constitute it vary with the physical geography and other circumstances of the country; and we should no more arrive at a knowledge of the character of the climate of the Mississippi valley by studying the climate of Italy or Russia, than we should at a knowledge of its fauna or its flora by studying the zoology or botany of corresponding regions in Europe or Asia. There is analogy everywhere, but as there is nowhere precisely the same combination of circumstances, so there are no countries in which the phenomena of climate are precisely identical. The climate of this great intermontane plane is subject to remarkable influences. In the first place, the country stretches, without a mountain barrier to deflect its winds, from the torrid to the frigid zone. Subtending it to the south is one of the warmest seas on the globe, and on the north it is bounded by the frozen ocean. On the east the Alleghany mountains shut it in from the winds of the Atlantic, and on the west it is sheltered from the Pacific ocean by the Rocky Mountains, which rise at some points into the regions of perpetual snow. At one time it is warmed by winds from the bosom of equatorial seas, under the genial influence of which frost yields at mid-winter; and again it is chilled by blasts sweeping down from the polar sea over fields of eternal ice. Thus, it is subject to all extremes of temperature, and it is also subject to every extreme of drought and humidity; but yet this climate of extremes and of change has its fixed laws, which only patience and industry, directed by a philosophical mind, are required to determine and expound. Dr. Drake has devoted nearly two hundred pages of his volume to the statistics of this interesting subject, and has here brought together a vast number of facts which he found scattered through the publications of the day. To many readers, it may be presumed, these details will appear tedious, and many it may be will not give them sufficient attention to understand them; but to any one desirous of studying our climate in the light of all its phenomena hitherto ascertained, they form the most ample and trustworthy source of information. It is possible that the subject is given more in detail than was demanded by the wants of most readers; but in its principal facts and general deductions every one must be supposed to feel some interest. The subject is evidently a favorite one with our author, and no part of his work is elaborated with more care than that relating to the climate of our valley.

Dr. Drake has taken a comprehensive view of the *hygiene* of the interior valley, and this will be found to be another important feature of his work. The third part of Book I is devoted to this subject, and embraces several points of the deepest interest. Population, its varieties and physiological characteristics, statistical physiology, the diet, clothing, and habits of its people, are some of the topics embraced in the discussion. They belong, as will be seen at a glance, to physiological and social etiology, and have a direct and important bearing upon disease. They are presented in this treatise, not in a general way, but with special reference to our own country, the facts and conclusions having been derived from the author's own observation. As climate varies incessantly with its modifying circumstances, so there are peculiarities in the hygienic condition of every people. The population of the great valley presents peculiarities in its social state which will strike the most superficial observer. To take but a single example—no people on earth exhibits such a commingling of races. We have four out of the five great varieties of the human species, and we have all the important branches of the variety which stands at the head of the race—the Caucasian. English, French, Irish, Scotch, Germans, Welsh, Hollanders, Swiss, Norwegians, Poles, Spaniards, Swedes, Italians, and Jews, with all their national traits, and all their peculiar social habits, have united here to form one great nation. For a century they have been mingling with each other and with the Aborigines of the country. In time, striking physiological and psychological developments must come from this union of races, for the process is going on every year with increasing activity; it has been going on among mankind in an accelerated ratio from the earliest ages. A law of increasing social amalgamation has governed the nations of men in all their migrations westward, from the sources of the Tigris and Euphrates to the banks of the Mississippi and the shores of our great lakes. The people who flourished at the head of the Mediterranean exhibited a character of much greater complexity than the nomadic tribes that wandered over the planes of Chaldea. When the tide of human life had reached Rome, its diversity was still further increased; but the Romans were a homogeneous people in comparison with the western nations that followed them in the march of civilization. The complexity has gone on deepening among the nations of Europe until some of them have become a mixture of many races; but there is still comparative simplicity among the people of the old countries. Upon our Atlantic shores a stream of population has been pouring in for centuries, from nearly every nation under the sun, and now it is flowing over into our valley—the union and living coalescence of the nations of mankind having thus been all along "in the direct ratio of time and distance from the birth-place of the species." But the world has been overrun, and this process of amalgamation is hastening to its final consummation; it is to be finished here. As remarked by Dr. Drake:

"The GREAT CENTRAL VALLEY OF NORTH AMERICA is the *last* crucible into which living materials, in great and diversified streams, can be poured for amalgamation. The double range of mountains which separate it from the Pacific Ocean, leave too little space for an empire on the shores of that sea; and the detached communities which may there grow up, will be but derivatives from the homogeneous millions, with which time will people the great region between the Appalachian and Rocky Mountains, which is thus destined to present the last and greatest development of society."

We proceed to mention another characteristic which places this treatise in the first class of medical writings—it is composed of original materials. While this remark is substantially true of all the introductory chapters of the work, it is emphatically so of that portion which relates specifically to disease. The article on *autumnal fever*, making the first part of Book II, consists of facts collected in our own valley. It is indigenous throughout—the work of the author, made up of his own observations, and those of his Western and Southern brethren. It is the first attempt, and an eminently successful one, to exhibit a full view of that disease, in all its forms, by the light of its phenomena as they occur in our country. No part of it is foreign; none is compiled, except from writers conversant with the disease as it exists here.

Now, we are not among those who have shown a disposition to exaggerate the importance of "sectional medicine." We are very far from believing, as some have contended, that in order to practise his profession successfully in the valley of the Mississippi, a physician must be educated in the valley of the Mississippi. Such a view of medicine we cannot help regarding as degrading to the science. In supporting it, its advocates have been guilty of much extravagance, and have put forth much that, not only will not stand the test of observation or sound logic, but that could hardly have been written seriously. At the same time, while we believe this, we take it to be a truth indisputable, that local soils and climates often engender peculiar maladies, and always modify their endemic disorders. Every country has its specific vegetables and animals. Corresponding zones of different countries exhibit striking analogies in their fauna and flora, but in only a few rare instances, specific identity. Something like the same, it can hardly be doubted, prevails with respect to disease; and if so, it follows that they who have been longest and most intimately conversant with the endemics of a country are best qualified, other things being equal, to write its medical history. No one will deny that a monograph on the plague, written by an observer on the banks of the Nile, would be more likely to exhibit a faithful and graphic picture of the disorder, than one written from hearsay or compiled from books. There is no one who would not prefer a history of yellow fever from the pen of a physician of New Orleans, who had been familiar with the epidemic in all its aspects, to one composed by a physician of Boston or Paris, who had never seen the disease, however learned and able he might be. Much of the value of Sydenham's works—we might say their chief excellence—consists in his faithful description of the epidemics which passed under his eye; and it will not be questioned that those portions of the writings of Dr. Rush which promise to live longest, are his admirable histories of yellow fever as it prevailed in Philadelphia.

This element of freshness and fidelity will impart an abiding interest to Dr. Drake's account of autumnal fever—the fullest, the most methodical and complete hitherto given of that disease. As the *type* disease of the valley it is treated of at much length, in all its varieties, and in reference to all the circumstances under which it originates. This fever is universally recognized as a disease of climate, but it was never before so clearly exhibited in its climatic relations, as it is in the work before us. Dr. Drake has traced it from zone to zone, showing that while its base is within the tropics its prevalence, owing to local causes, is sometimes greater at points farther north, but that it declines with much uniformity under a decreasing summer temperature, until finally it ceases to be an endemic of the country. For example, at the military post at Key West, north latitude 24° 33′ the annual number of attacks for a series of years among 1,000 men, is shown to have been 190; while at Fort Brooke, in north latitude 27° 57′ the number was 849; at Fort King, 29° 12′, 1,387; and at Fort Jackson, 29° 29′, 1,600. At the New Orleans barracks, the number of attacks, among a like number of men, was 544; while at Fort Wood, nearly a degree further north, the number was 1,063; at Fort Pike, north latitude 30° 10′ the number of attacks was 232, and at Baton Rouge, some minutes further north, 824; at Fort Mitchell, north latitude 32° 19′ the number was 225, and 1,265 at Fort Towson, in north latitude 33° 51′, and 1,161, at Fort Smith, two degrees further north. But at Fort Howard, north latitude 44° 40′, the number declines to 84, at Forth Snelling, nearly the same latitude, to 62, at Fort Brady, one degree north, to 44, and in Lower Canada, in about the same latitude, to 27. Finally, from all the observations made by Dr. Drake, it would appear that the geographical limits of autumnal fever are reached, at the level of the sea, about north latitude 47°.

It is a curious and interesting fact observed by naturalists, that ascending forest vegetation corresponds with the distribution of trees over the temperate zone; or, in other words, that elevation is equivalent to recession from the equator. The order in which the families of trees appear as we go north over the temperate zone, is the order in which they are presented as we ascend the sides of mountains—first the Juglandeæ and Amentaceæ, then the Acerinæ, and finally, towards the summits, the Coniferæ. Nut-bearing trees are proper to temperate regions; maples predominate in climates more rigorous, and pine forests are the last that disappear under the increasing severity of northern skies. In regard to febrile diseases the same law seems to hold; latitude and elevation bring about the same results. Yellow fever is an endemic of equatorial regions; it is also occasionally seen in the temperate zone, at the level of the sea. As we recede from the equator, and rise above the level of the sea, we leave it behind us. Philadelphia and New York were once subject to it, and they now suffer with other forms of autumnal fever, but in the mountainous villages in the same latitudes, remittent fever prevails only to a limited extent, and intermittent fever is nearly unknown. All the favoring circumstances of marsh and decaying organic matter fail to originate autumnal fever at the forty-seventh degree of north latitude; and the same combination of causes is insufficient to its development on the summits of elevated mountains.

The work before us contains many facts illustrative of this law, one of which is so striking that we shall here present it in detail. The basin of the Genesee river, in latitude 43° N., furnishes all the geological elements necessary to the origin of autumnal fever, and the testimony of medical men is that it has been visited, in times past, by fevers of a malignant character. "Genesee fever" was, in the earlier years of the settlement of the country, a familiar appellation. With the progress of agriculture, the disease has become less frequent, but sporadic cases continue still to occur, every year, in the depths of Rochester, the largest city in that basin. The Genesee river descends from the mountains in a southernly course towards the Lake. Its head streams issue, with those of the Cataraugus, Alleghany, and Susquehanna, from a summit-level where frost occurs regularly in June and August, and not unfrequently in July. In visiting this region, Dr. Drake "saw many fields of Indian corn that had been frost-bitten on the night of the third of August;" and in passing north to Lake Ontario, he met "the scythemen, who had completed the harvest in the country below, advancing south, and mounting on the higher level, to continue their labors."

"In ascending this mountain slope, although we go directly south, we find that the fevers which prevail on the flats below, and down to the shores of Lake Ontario, get less and less. They prevail more along the Genesee River, and for a short distance up its tributaries, than elsewhere; but at length are almost unknown in every kind of locality, even the most paludal. At the village of Pike, on the banks of the transparent West-Koy, six or eight miles from the Genesee River, and at an elevation (by estimate) of twelve hundred feet above the sea, Doctor Capron, who had resided there twenty-eight years, assured me they were unknown; adding, that the stream abounded in *trout*, a certain sign of exemption from that disease. Cases of remittent fever, however, now and then occur. Doctor Minard, of the same village, confirmed these statements; but informed me that both intermittents and remittants occur, to some extent, near the junction of the West-Koy with the Genesee, and, also, on the corresponding portion of a neighboring tributary, Cole Creek.

"The summit-level on which the Genesee, in common with the Alleghany River, originates, lies between 42° and 42° 30′ N. latitude, and has an elevation varying from thirteen hundred to sixteen hundred feet. It is about one degree farther south, and twelve hundred feet higher than the shores of Lake Ontario, between the mouths of the Niagara and the Genesee Rivers. Now, an autumnal fever, especially the intermittent variety, is the principal endemic of those shores, but almost unknown on this platform; and the difference must be ascribed to altitude, as swamps, streams, and organic matter abound in this region."

Four fifths of this large volume, or about seven hundred pages, are devoted—and in our view judiciously devoted—to Etiology—first, and most largely, to *Topographical and Hydrographical Etiology*, second, to *Climatic Etiology*, and third to *Physiological and Social Etiology*. Some readers, doubtless, will weary of the protracted details given under these several heads; but what subject, we desire to know, has higher interest for the physician than general etiology—the study of man's relations to the earth, to the air, and the food, drinks, and clothing by which he subsists? It is the philosophy of health—of living long and comfortably. The efficiency of the healing art, we have the fullest persuasion, is destined to a great improvement in the line of Therapeutics, but, unquestionably, among the greatest benefactions that medical science has yet conferred upon the race, have been the gifts of Hygiea. Very much remains to be done in perfecting our remedial measures, and our faith is great in the future of curative medicine; but we believe that not less will be accomplished for the amelioration of human health and the extension of human life, by Etiology—the study of the diversified causes of disease. We have thus far found it much better to prevent than to cure small pox. When we shall have ascertained the cause of cholera, and all the circumstances that go to develop it and give it intensity, who can doubt that we shall achieve more by averting or removing it than we are likely ever to do by all the resources of the apothecary? We have already taken an important step towards the prevention of autumnal fever in having ascertained with so much certainty the conditions which give it origin. How much has been done to dry up the sources of disease and increase the average duration of human life in cities by attention to the principles of hygiene in draining and ventilation, so that the mortality of some of them is now not so great, in times of pestilence, as it was, in centuries past, during the healthiest seasons! How much greater will be the chances of life and how much higher the sum of its enjoyments, when the laws of hygiene shall have come to be generally understood and respected!

This is evidently Dr. Drake's way of thinking; he is one of the writers on practical medicine who have wisely given to Etiology the prominence it deserves. It has been his habit to take enlarged and philosophical views of medicine. We remember well a course of lectures delivered by him on the practice of physic to which we listened with great pleasure, a good many years ago, and which we admired especially for its deep and broad foundation laid in general pathology. If our memory is correct, he devoted more than a fourth of his course to preliminary views; nor shall we soon forget how irksome—how "stale, flat, and unprofitable"—they proved to not a few of his auditors. Day after day we remember to have seen assiduous students repairing to his lecture-room, note-book in hand, prepared to write down his teachings, but after listening in vain for the first fifteen minutes of the lecture for something *practical*—for some formula or specific—they would shut up their books in despair and sit for the remainder of the hour in listless vacuity. If his book should fall into the hands of any readers as wanting in taste for the general principles of medicine, we can hardly hope to have their concurrence in our estimate of its merits.

We believe it will not be denied that the range of professional studies, in our country, however liberal it may be in theory, is practically too limited, and that the tendency of the times is to limit it more and more. We are disposed to make medicine too technical—too much a thing of recipes. The period of study has been wonderfully abridged, and there is danger that we are also abridging the extent of our studies. We hurry through the elements to reach practical medicine. The science is not properly appreciated, and we attach undue importance to the art, the consequence of which is that the former is not thoroughly mastered by pupils, and is generally neglected and soon forgotten by practitioners. Now, we would ask, is not this one cause of that want of elevation in the profession of which we hear so much complaint in our day? Would it not be well if students were required to add some other elementary branches to anatomy, physiology, and chemistry, and to make themselves proficients in these before entering upon the study of the practice? Would not the benefit be great of making Etiology a part of the course for students? Besides the practical knowledge derived from it—the acquaintance with the sources of our physical maladies gained, and the means of warding them off—it would favor habits of observation, the most valuable in a physician, and inspire more enlarged and liberal views of medicine. The effect of such an extended course of study would be to make medical philosophers, instead of, what is too often the result of the present system, mere routine practitioners.

But we must leave these general reflections, which have been suggested by the perusal of Dr. Drake's work, and proceed to give a more particular account of its varied contents. As we have already remarked, the first seven hundred pages of the volume relate to Etiology. First, the boundaries, area, and aspects of the valley are given, after which follows a view of its hydrology. The hydrographical system of this region embraces seventeen axes, to which nearly all the rivers of the continent may be referred. That which gives origin to the Mississippi river is first in importance, for in it rises also the no less remarkable stream which, in its course, expands into the great northern Lakes, and is precipitated over the celebrated Falls of Niagara; to reach the sea under the name of the St. Lawrence, having been, at various points, the St. Louis, the St. Mary's, the St. Clair, the Detroit, and the Niagara river. The Mississippi lies in the trough or synclinal axis of the valley, through a distance, following its sinuosities, of nearly three thousand miles, or more than sixteen degrees of latitude. This trough, Dr. Drake supposes, was excavated by a wide and deep current, which flowed across the continent from the north, and of which Lake Traverse and Big Stone Lake are but chasms or hollows, left filled with water when that river ceased to flow. This great aqueduct is separated from those which drain the valley north and east by so slight an elevation that, in times of flood, canoes have passed from one into the other. Dr. Drake gives the following illustration of the fact:

"Two voyagers might start from the Gulf of Mexico, in the latitude of twenty-nine degrees north; and ascending the Mississippi to the mouth of the Illinois river, one of them might take the course of that stream; pass into the Lakes, over an elevation of six hundred feet; descend the St. Lawrence; and make his exit into the Atlantic Ocean, upon the fishing banks of Newfoundland; twenty degrees north and thirty degrees east of his place of departure. The other, continuing up the Mississippi and over the Lake Traverse summit level, at the hight of nine hundred and seventy-five feet, would descend into Hudson Bay; whence he might pass into the polar seas, amid perpetual ices; after having traversed more than forty degrees of latitude."

A notice of the geology of the great valley follows the description of its geographical and hydrographical system, but having already given a sufficiently full account of this part of the work we pass on to other topics.

The valley is subdivided into four great basins; one containing the Mississippi river and its tributaries, which Dr. Drake denominates the Southern, or Mexican; another embracing the Lakes and the St. Lawrence, which he calls the Eastern or St. Lawrence; a third which holds the waters flowing into Hudson Bay, the Hudson basin; and the last, the Polar basin, which includes the northern sea coast of the continent, from Baffin Bay to the Rocky Mountains. The first is the most important in a medical point of view; it stretches quite across the valley, and belongs chiefly to the United States. The St. Lawrence is set into one side of the valley. Next to the Southern, it contains the largest population, and is most interesting to medical men; it is divided almost equally between the United States and Great Britain. The Hudson, like the Mexican, extends across the valley; in population it ranks third; it lies principally within British jurisdiction, but a portion of it projects into the United States. The whole of the Arctic basin appertains exclusively to Great Britain. Its inhabitants are the Esquimaux—the Mongolian variety of the denizens of the great valley—and are remarkable for living in a lower temperature than any other known portion of the human race.

Having given this general description of the region, Dr. Drake next goes on to present an analysis of the several topographical basins, beginning with the Southern. It would be in vain to attempt to condense any further what is already given in the briefest form compatible with clearness; we shall not therefore pretend to make an abstract of his topography, but merely notice a few of the conclusions at which the author arrives under this head.

In closing his description of the Bay of Pensacola he States the following facts bearing upon the origin of fever:

"The yellow fever has been several times prevalent in the town, among the shipping, and at the Navy Yard; but the number and malignity of its invasions bear no comparison to its severe visitations of Mobile and New Orleans.

"Of autumnal intermittent and remittent fever, it will be proper to speak more extensively. From the forts to the town of Pensacola inclusive, (all on the west side of the Bay,) although there are some swales and small swamps or ponds among the sand dunes, and some narrow tracts of salt marsh, there are, as we have seen, no deposits of silt; and the organic matters accumulated in the wet or paludal spots, are chiefly those which belong to the pine forest. Now the inhabitants of this range of coast have for a long time enjoyed an exemption from autumnal fever, remarkable for a southern locality. The town of Pensacola has even been resorted to as a summer residence by citizens of Mobile and New Orleans. When, however, we ascend the same coast, about ten miles above the town, to the estuary of the Escambia River, we find a state of things entirely different. The silt brought down by that stream, has filled, as we have seen, a large portion of the western head of the Bay, and thus generated a marsh, several miles in width, near which the settlers have been fatally scourged by autumnal fever; although they escaped yellow fever when it prevailed in the town and Navy Yard below."

He then quotes from Lind the history of an attempt to settle this devoted locality eighty years ago: Lind says:

"In the year 1766, sixteen French Protestant families, consisting of sixty persons, were sent, at the expense of the English government, to West Florida. The ground allotted for their residence was on the side of a hill, surrounded with marshes, at the mouth of the river Scambia.* These new planters arrived in winter, and continued perfectly heaithy until the sickly months, which in that country are those of July and August. About that time eight gentlemen (from one of whom I received this account) went to this new settlement to solicit votes for the election of a representative in the general assembly of the province; by remaining but one night, every one of them was seized with a violent intermitting fever, of which the candidate for becoming the representative, and another of their number, died. The next day seven other gentlemen came upon the same business to this unhealthy spot; but, by leaving it before night, they escaped the sickness, and all continued in perfect health. Among the French settlers, during these two months the annual fever of the climate proved so fatal on this unwholesome spot, that of sixty persons fourteen only survived; and even those who remained alive, in the September and October following, were all in a very ill state of health; not one of them had escaped the attack of the fever, and most of them died within a few months afterward, from the injury it had done to their constitutions."

Dr. Drake continues—

"No other settlement in this locality seems to have been attempted for a long time afterward. At length, in the year 1832 or 1833, a new attempt was made, by laying off a town, to be called Florida, on the eastern side of the estuary, in the edge of the pine woods. All the pine terraces of the south are proverbially free from autumnal fever; but here the pine lands lie to the leeward, while extensive silt marshes spread out to the windward. Between twenty and thirty wooden houses were bult, and tenanted by as many families. Their history, as given by Dr. Hulse, the intelligent and reliable surgeon of the Naval Hospital, and by Mr. Innerarity and Mr. Kelly, old and respectable citizens of Pensacola, may be told in a few words. Year after year, while the inhabitants of the coast below remained healthy, they were assailed by au-

*Escambia.

tumnal fevers of the most malignant character; the spot was at last called a 'Graveyard;' and being abandoned by those who survived, I found, on passing through it in 1843, but two families remaining.

"These well-ascertained facts have so important a bearing on the origin of the autumnal fever, that I have considered them worthy of circumstantial detail. The heat and moisture of the lower and upper portions of this little Bay are the same; but while the former has only a few limited tracts of pine marsh, the latter includes extensive deposits of silt and organic matter; and to them, I think, we are bound to attribute the fatal insalubrity which has been described."

Speaking of the Pine Woods he says—

"Such are the celebrated *Pine Woods*, to the protecting influence of which the people of New Orleans and Mobile commit themselves for safety, in yellow fever seasons; expecting to enjoy an equal immunity from intermittents and remittents. Thus, in the region we are describing, the sweet gum and cypress, with their festoons of moss, are the symbols of deep soil, foul surface, impure water, vegetable decomposition, and fevers; while the long-leafed pine, symbolizes sterility, dryness of surface, gushing springs of pure water, and sound health."

He concludes his account of the impurities of the Mississippi river with the following remarks—

"The salubrity of the Mississippi water, or that of the Missouri which imparts the character of turbidness, is not an open question. From St. Louis to New Orleans, the testimony of the population on its banks, and of those who spend a great part of their lives upon it as watermen, is unequivocally in its favor. Many persons drink it before its suspended materials have subsided, and seem to prefer it to that which has been rendered transparent by time or art. That it produces some effects on the system, which transparent water, from wells and springs, and our other rivers, does not, is an established popular opinion. It is even regarded by many persons as being, to a certain extent, medicinal, and especially adapted to the cure of chronic functional disorders of the stomach, bowels, and liver—an opinion in which I am disposed to concur. That its daily use averts some forms of disease, may be admitted as probable; but precise observations on all these points are wanting; and I shall dismiss the subject with the presentation of two facts, in which, I trust, the reader will take a pleasant interest. *First:* Professor Bailey, after observing its numerous shoals of microscopic animalcules, expresses the opinion, that they are sufficiently abundant to render the water somewhat *nutricious. Second:* In his Letters on Louisiana, written in the year 1751, Captain Boissu informs us, that the Mississippi water has the property of contributing to the '*fecondite des femmes!*' "

The earlier readers of this Journal will remember an elaborate paper by Dr. Cartwright on the salutary influence of the Jussieua grandiflora in preventing malarious fevers. Dr. C. ascribes the exemption of large districts of country in Louisiana to the growth of this plant. In regard to his hypothesis Dr. Drake makes the following remarks:

"1. The 'coasts,' as they are called, or banks of the Mississippi, from New Orleans to the outlets of Bayou La Fourche and Bayou Plaquemine, (lying nearly north of the region where the Jussieua is supposed to destroy the cause of autumnal fever,) are equally exempt from that disease, and equally abound in aged Creoles, although there are no lakes and no Jussieua; but the river is on one side, and cypress swamps are on the other. I was prevented from visiting the district where the Jussieua grows, but traveled on the coasts.

"2. If we examine the locality which the Jussieua overspreads by the facts furnished by Darby, Cartwright, and others, we do not find that it abounds in those elements to which malaria is generally ascribed. Lakes, *in themselves*, are certainly not of that kind. The bayous, these writers inform us, are natural canals, several feet deep, in which the tides of the Gulf daily librate. The central belts between them, called shaking prairies, are lakes bridged over with matted plants; for fish may be caught by perforating their trembling crusts. The narrow zone of palmetto swamp, which skirts the shaking prairie, is nearly destitute of annual vegetation; and the leaves of that shrub are perennial and most difficult of decomposition. Then comes the narrow tract of cypress swamp, densely overshadowed, and equally devoid of herbaceous vegetation. To this succeeds the belt of cane-brake and deciduous forest trees, so dense that a bird cannot fly through, nor a sunbeam reach the surface, which of course can sustain no succulent annual plants; then a belt of live oak, a quarter of a mile in breadth; and lastly, the narrow zone of long-cultivated, arable land, terminating in the lake or bayou. Compared with the other varieties, the tillable portions do not make a third; and they are no longer subject to the inundations of the Mississippi, which for ages could not have thrown upon them, by its overflowings, any great amount of organic matter; as most of it, in so long a voyage, is either deposited above, or decomposed, or so comminuted, that it remains suspended until the inundation gradually sinks into the Gulf. Thus, *a priori*, we should not expect to see as much autumnal fever in that region, as in those further up the river.

"3. Dr. Cartwright ascribes the transparency of the lakes in which the Jusseiua floats, to the action of that plant; but may we not, as plausibly, say, that it prefers clear to turbid waters? With these facts before us, we should, I think, regard the preventive powever of the Jussieua as an open question."

The Balize was visited by Dr. Drake and his account of what he ascertained respecting its diseases is communicated in the following paragraphs:

"The prevailing disease at the Balize, and the South West Pass, is intermittent fever, generally of the tertian type, and mild in its character, with a tendency in the patients to relapse. Doctor Van Antwerp had seen only two malignant cases. Some immigrants have lived there several years before they sickened. Mrs. Anderson, who had resided there longer than any other individual, thought the disease much less frequent than formerly. Doctor Van Antwerp had noticed a considerable number of diseased spleens, but very little neuralgia or dropsy, consequent on the fever. Remittents appear to be decidedly rare, and the same is true of yellow fever, notwithstanding almost every vessel from Havana and Vera Cruz enters through this Pass, and is visited by the pilot and boarding officer. Doctor Van Antwerp arrived in October, 1839, when the fever was extensively prevalent around the shores of the Gulf, including, of course, New Orleans; and in 1841 and 1842, it was prevalent in that city and some other places; still he had seen only four cases; one of which occurred in a person from New Orleans; two others seemed to have originated in the village, and the fourth occurred in an oysterman, who declared he had not been at New Orleans. These cases occurred in different years. Eruptive fevers are exceedingly uncommon, and chronic cutaneous disorders seldom show themselves. The itch is said to lose its contagiousness, and at length cease. The summer gastro-intestinal affections, such as cholera morbus, cholera infantum, and dysentery, especially the two former, are unfrequent. Pulmonary inflammations of all kinds are quite as uncommon. Doctor Van Antwerp was induced to remove from the State of New York to the Balize, on accout of his liability to pulmonary catarrh, which his residence at the latter place has nearly removed. Croup is almost unknown. Of nervous diseases, convulsions of children are more frequent than all the rest. Doctor Van Antwerp had seen nine cases, five of which proved fatal. The number of children among whom they occurred, was about forty—the time, three and a half years. The children who suffered, were not, as it is termed in the nursery, 'within the month.' Another disease of frequent occurrence is rheumatism, which is generally subacute or chronic, and falls especially on the pilots who are greatly exposed. Consumption is said to be rare. Captain Taylor, who had resided there sixteen years, could recollect but three cases, and the patients had all arrived there with the disease in its forming stage. Nevertheless, his wife, who many years before had left the state of New York, was evidently falling into that disease, and two of his children, born at the Balize, had strumous swellings and abscesses of the neck." Y.

*(To be continued.)*

